# Is the axial length a risk factor for post-LASIK myopic regression?

**DOI:** 10.1007/s00417-020-04990-4

**Published:** 2020-10-31

**Authors:** Amr A. Gab-Alla

**Affiliations:** Faculty of Medicine, Ophthalmology Department, Suez Canal University, Ring Road, Ismailia, Egypt

**Keywords:** Axial length, LASIK, Myopia, Correlation, Regression

## Abstract

**Purpose:**

To assess the relationship between the axial length and post-LASIK regression in myopic patients.

**Methods:**

This is a retrospective case series study conducted at a private eye centre, Ismailia, Egypt. The clinical records of the patients, who experienced LASIK to correct myopia from January 2016 to January 2018, were analysed for myopic regression. The patients were operated on, examined, and followed-up 1 year by one surgeon (AAG).

**Results:**

This study included 1219 patients (2316 eyes) with myopia. Mean ± SD of pre-operative spherical equivalent (SE) was − 4.3 ± 2.1D, range (− 0.50 to − 10.0D). Mean ± SD age of the patients was 26.4 ± 6.8 years, range (21 to 50 years). Male to female ratio was 30.5 to 69.5%. The cumulative incidence rate of myopic regression according to the medical records of the patients was 25.12% (582 eyes out of total 2316 eyes) along the 2 years of this study (12.6% per year). Of the total patients, 14.94% had pre-operative high myopia, 35.84% had pre-operative moderate myopia, and 49.2% had pre-operative low myopia. Of the patients with myopic regression, 52.6% had pre-operative high myopia, 34% had pre-operative moderate myopia, and 13.4% had pre-operative low myopia. The mean ± SD of the axial length of the patients with myopic regression was 26.6 ± 0.44 mm, range (26.0 to 27.86 mm), while the mean ± SD of the axial length of other patients with stable refraction was 24.38 ± 0.73 mm, range (22.9 to 25.9 mm) (*t* test statistic = 69.3; *P* value < 0.001).

**Conclusions:**

Pre-operative high axial length increases the risk of myopic regression after LASIK.
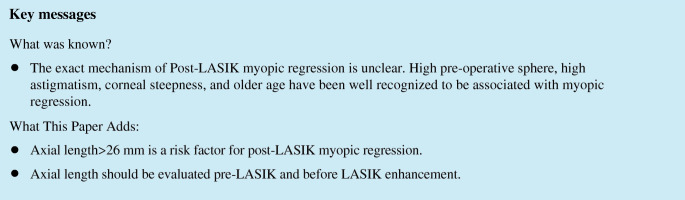

## Introduction

Post-laser in situ keratomileusis myopic regression can be defined as gradual, incomplete, or complete loss of primary correction that limits the efficiency, predictability, and long-term stability of laser in situ keratomileusis (LASIK) [1].

Although LASIK techniques and surgeon experience have improved over the past 20 years resulting in better outcomes, myopic regression after LASIK is inevitable, and the exact mechanism of it is unclear and under-explored [2]. Previous studies had recorded some regression-related factors before and after LASIK and suggested a forward shift of the cornea as an explanation of myopic regression [3–5]. Patel et al. [6] recorded a myopic regression rate of 16% along the 3-month follow-up period of their study. Hersh et al. [7] recorded a 10.5% incidence of myopic regression in their study (3-year follow-up period). They stated that high primary corrections, astigmatism, and old age were significant risk factors for it. Liu et al. [8] recorded a rate of 21% myopic regression along 5 years of their study. Randleman et al. [9] recorded a 6.3% enhancement rate along 1-year follow-up period and stated that patients with high myopic errors were more expected to have enhancement. In long-term studies, Alio et al. [10] recorded retreatment rates ranging from 20 to 27% in their study with 10 years follow-up.

Another factor that might influence the post-LASIK myopic regression is the axial length of the eye. So, this study aimed to assess the relationship between refractive regression and the axial length in myopic patients.

## Patients and methods

This is a retrospective and case series study that included patients who experienced LASIK to correct myopia from January 2016 to January 2018, at a private eye centre, Ismailia, Egypt.

Inclusion criteria were age over 21 years, intraocular pressure (IOP) less than 21 mmHg, central corneal thickness (CCT) > 500 μm at the thinnest point and the calculated residual stromal bed after treatment > 60% of the total corneal thickness, a regular corneal topography pattern (Sirius, CSO, Florence, Italy), and no history of diabetes mellitus, autoimmune diseases, or earlier ocular surgeries. Patients with insufficient follow-up were excluded from the study.

Pre-operatively, patients experienced standard eye examinations, including slit-lamp examination, indirect fundoscopy, and refraction (cycloplegic and manifest and presented as spherical equivalent − 0.5 to − 10.00D), intraocular pressure (IOP) by applanation tonometry and ocular response analyser (ORA), and axial length (Lenstar, HAAG-STREIT, USA).

Laser in situ keratomileusis was performed using 500 kHz Amaris excimer laser (Schwind eye-tech-solutions, Kleinostheim, Germany). Corneal flaps with superior hinge were cut with Moria M2 microkeratome (Moria, Antony, France). The optical zones ranged from 5.8 to 7.0 mm in diameter. The patients had routine procedures. All surgeries targeted distance vision.

Post-operatively, the patients with stable refraction were examined at the 1st day; 1st week; 1st, 3rd, and 6th months, and 1 year after the surgery. Patients with myopic regression were examined monthly until enhancement. The patients were assessed by complete ocular examinations. The main outcome measures were refraction (cycloplegic and manifest) and corneal topography. Patients with topographic signs suggesting corneal ectasia, under correction, corneal haze, or other complications were excluded from the study. The patients with myopic regression were only included in this study. Patients were encouraged to return after surgery for an examination if their vision declined. Enhancement surgery was offered when needed (patient dissatisfaction with the visual result). Enhancement was done by re-lifting the flap and treating the stromal bed for all patients with myopic regression (no dropout). The patients were operated on, examined, and followed-up by one surgeon (AAG).

### Data collection

The medical records of the patients with refractive regressions were reviewed. The demographic and pre-operative data were collected (age, sex, pre-operative refraction, axial length, keratometric reading, and corneal topography). In this study, refractive myopic regression will be considered as a myopic shift of **≥** 0.5D in cycloplegic refraction after LASIK full correction.

The tenets of the Helsinki Declaration were followed in this study. It was reviewed and agreed by the Faculty of Medicine, Suez Canal University research ethics committee. Informed consent was not necessary for the analysis of the medical records due to the retrospective design of the study and the large sample size.

### Statistical analysis

All data manipulation and analysis were performed by the Statistical Package for the Social Sciences (SPSS) version 25 (IBM Corporation, NY, USA). Parameters of the study groups were presented as frequencies and percentages or mean values and standard deviations. The student’s *t* test was used to compare the differences between means in the groups. Differences between frequencies in the groups were compared by the Chi-square test or Fisher’s exact test (if > 20% of expected values were less than 5). To compare the difference in the mean measurements between the sub-groups of myopia, one-way ANOVA was performed. Shapiro-Wilk’s test was used to test for data normality. Graphs were performed with GraphPad Prism (version 5.00 for Windows, GraphPad Software, La Jolla, CA, USA). A *p* value < 0.05 was considered statistically significant.

## Results

This study included 1219 patients (2316 eyes) with myopia. Mean ± SD of pre-operative spherical equivalent (SE) was − 4.3 ± 2.1D, range (− 0.50 to − 10.00D). Mean ± SD age of the patients was 26.4 ± 6.8 years, range (21 to 50 years). Male to female ratio was 30.5 to 69.5%. Mean ± SD of the K min was 43.3 ± 1.7D, range (38.5 to 48.9D), and of the K max was 44.8 ± 1.2 D, range (39.9 to 49.6D). The cumulative incidence rate of myopic regression according to the medical records of the patients was 25.12% (582 eyes out of total 2316 eyes) along the 2 years of this study (12.6% per year). The characteristics of all patients are presented in Table 1.

**Table 1:** The characteristics of all patients (pre-operative)

The characteristics	All eyes
Eyes: *n*	2316
Sex: *n*, (%)	
Female	1610 (69.5%)
Male	706 (30.5%)
Age (yrs)	
Mean ± SD	26.4 ± 6.8
Range	(21 to 50)
SE (D)	
Mean ± SD	− 4.3 ± 2.1
Range	(− 0.5 to − 10.0)
*K* min (D)	
Mean ± SD	43.3 ± 1.7
Range	(38.5 to 48.9)
*K* max(D)	
Mean ± SD	44.8 ± 1.2
Range	(39.9 to 49.6)

*n* number, *SE* spherical equivalent, *K min* minimum keratometric power, *K max* maximum keratometric power, *yrs* years, *D* diopter, *SD* standard deviation

The total myopic eyes were classified according to the pre-operative refraction into three sub-groups: low myopia > − 3.0D, moderate myopia − 3.0 to > − 6.0D, and high myopia **≤** − 6.0D. In total, 49.2% had pre-operative low myopia with mean ± SD of − 2.3 ± 0.7, 35.84% had pre-operative moderate myopia with mean ± SD of − 4.9 ± 1.2, and 14.94% had pre-operative high myopia with mean ± SD of − 8.4 ± 1.8. The characteristics of each group are presented in Table 2.

**Table 2:** The total myopic eyes classified according to the pre-operative refraction into three sub-groups

	Total myopic eyes (*n* = 2316) *n* (column %)			*P* value
	Low > − 3.0D	Moderate − 3.0 to > − 6.0D	High < − 6.0D	
Number (%)	1140 (49.2%)	830 (35.84%)	346 (14.94%)	–
Age (yrs)				0.0111*
Mean ± SD	26.4 ± 6.2	26.6 ± 6.6	27.6 ± 7.4	
Range	(21 to 50)	(21 to 50)	(21 to 50)	
Sex: *n*, (%)				0.022*
Male	480 (42.1%)	312 (37.6%)	158 (45.7%)	
Female	660 (57.9%)	518 (62.4%)	188 (54.3%)	
Eye: *n* (%)				0.790
Right	574 (50.4%)	408 (49.15%)	168 (48.6%)	
Left	566 (49.6%)	422 (50.85%)	178 (51.4%)	
SE(D):				< 0.001*
Mean ± SD	− 2.3 ± 0.7	− 4.9 ± 1.2	− 8.4 ± 1.8	
Range	(− 0.5 to − 2.75)	(− 3.0 to − 5.75)	(− 6.0 to − 10.0)	
*K* min (D)				< 0.001*
Mean ± SD	43.3 ± 1.3	43.7 ± 1.4	43.9 ± 1.6	
Range	(38.5 to 47.9)	(38.7 to 47.8)	(40.0 to 48.1)	
K2 max(D)				< 0.001*
Mean ± SD	44.4 ± 1.7	44.7 ± 1.4	45.2 ± 1.5	
Range	(40.2 to 49.0)	(41.4 to 49.6)	(42.1 to 49.6)	

*SE* spherical equivalent, *n* number, *yrs* years, *SD* standard deviation, *D* diopter, *K min* minimum keratometric power, *K max* maximum keratometric power

*Statistically significant

Eyes with post-LASIK myopic regression were classified according to their pre-operative refraction into three sub-groups:

- 13.4% had pre-operative low myopia with mean ± SD − 2.75 ± 0.1D.
- 34% had pre-operative moderate myopia with mean ± SD − 4.43 ± 0.73D.
- 52.6% of the patients had pre-operative high myopia with mean ± SD − 7.91 ± 1.45D.

The characteristics of those patients are presented in Table 3.

**Table 3:** The characteristics of eyes with Post-LASIK myopic regression

	Patients with myopic regression (*n* = 582) *n* (column %)			*P* value
	Low myopia > − 3.0D	Moderate myopia − 3.0 to > − 6.0D	High myopia ≤ − 6.0D	
Number (%)	78 (13.4%)	198 (34%)	306 (52.6%)	---
Age (yrs)				
Mean ± SD Range	21 ± 0.1 (21 to 29)	24.6 ± 5.1 (21 to 36)	25.65 ± 6.8 (21 to 39)	0.166
Sex: *n*, (%)				
Male Female	28 (36%) 50 (64%)	124 (62.6%) 74 (37.4%)	108 (35.3%) 198 (64.7%)	< 0.001*
Eye: *n*, (%)				
Right Left	40 (51%) 38 (49%)	74 (37.4%) 124 (62.6%)	200 (65.4%) 106 (34.6%)	< 0.001*
SE(D):				
Mean ± SD Range	− 2.75 ± 0.1 (− 2.50 to − 2.75)	− 4.43 ± 0.73 (− 3.50 to − 5.5)	− 7.91 ± 1.45 (− 6.25 to − 10.0)	< 0.001*
K min(D)				
Mean ± SD Range	40.07 ± 0.12 (39.97 to 40.2)	41.49 ± 1.42 (39.82 to 44.01)	43.52 ± 1.35 (41.6 to 45.23)	0.001*
K max(D)				
Mean ± SD Range	42.06 ± 0.08 (41.99 to 42.13)	43.34 ± 1.35 (41.7 to 45.7)	44.96 ± 1.24 (42.4 to 46.7)	0.001*

*SE* spherical equivalent, *n* number, *yrs* years, *SD* standard deviation, *D* diopter, *K min* minimum keratometric power, *K max* maximum keratometric power

*Statistically significant

**Fig. 1:**
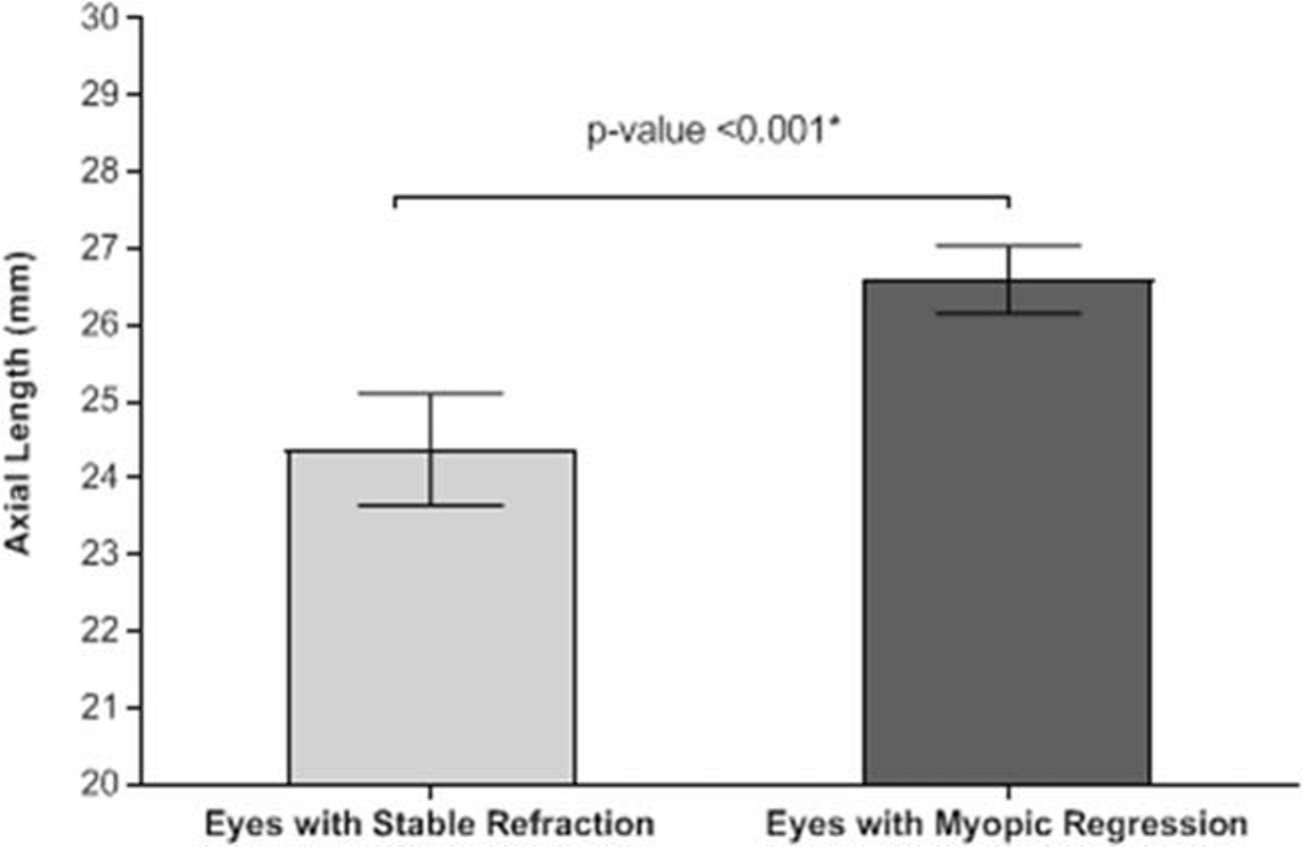
Mean of the axial length in eyes with stable refraction and eyes with myopic regression. There is a statistically significant difference between the two groups (*P* < 0.001)

**Fig. 2:**
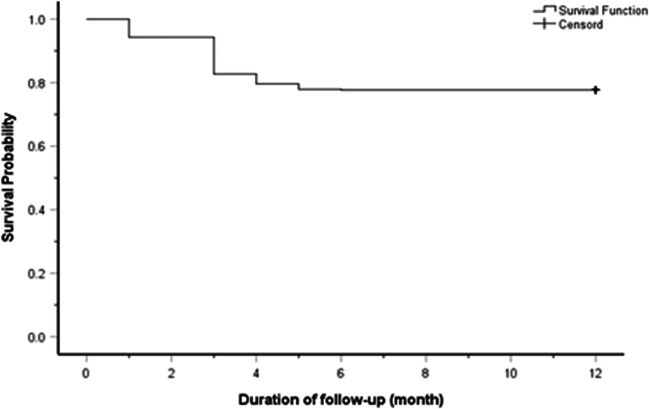
Survival curve of the total eyes shows that all myopic regression occurred during the first 6 post-operative months, with a mean survival time among eyes with myopic regression of 3.0 ± 1.0 months

**Fig. 3:**
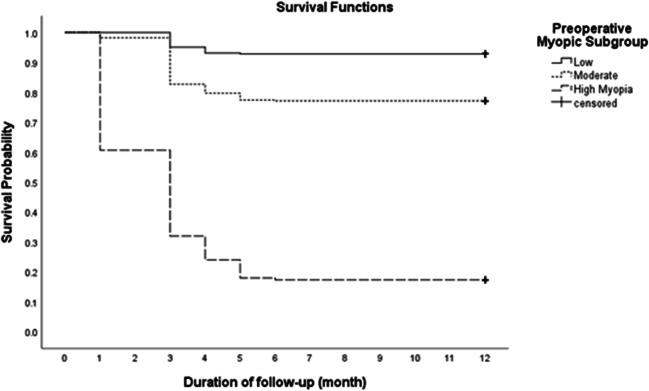
Survival curve of post-LASIK myopic regression according to the pre-operative myopic sub-groups

The mean ± SD of the axial length of the patients with myopic regression was 26.6 ± 0.44 mm, range (26.0 to 27.86 mm), while the mean ± SD of the axial length of other patients with stable refraction was 24.38 ± 0.73 mm, range (22.9 to 25.9 mm) (*t* test statistic = 69.3; *P* value < 0.001). The characteristics of the eyes with post-LASIK stable refractions and the eyes with myopic regression are presented in Table 4 and Fig. 1. The mean ± SD of the time between initial correction and regression was 3.0 ± 1.0 months; Fig. 2 shows the survival curve of the total eyes. About 3% of all studied eyes developed myopic regression at the 1st month, compared to 5.8%, 1.6%, 0.9%, and 0.1% at 3rd, 4th, 5th, and 6th month, respectively. Figure 3 shows the survival curve of post-LASIK myopic regression according to the pre-operative myopic sub-groups. There were significantly unequal survival distributions for the different levels of pre-operative myopic sub-groups (Log Rank test = 590.3; *P* value < 0.001). Compared to eyes with pre-operative low myopia, eyes with pre-operative high and moderate myopia had significantly less mean survival time (9.99 and 3.99, versus 11.38 months). Further, eyes with pre-operative high myopia developed myopic regression at earlier months of the follow-up period, compared to eyes with moderate and low myopia (1st versus 3rd month).

**Table 4:** Characteristics of the eyes with post-LASIK stable refractions and the eyes with post-LASIK myopic regression

	Total eyes (*n* = 2316) *n* (column %)		*P* value
	Eyes with stable refraction (*n* = 1734)	Eyes with myopic regression (*n* = 582)	
Axial length			< 0.001*
Mean ± SD	24.38 ± 0.73	26.6 ± 0.44	
Range	22.9 to 25.9	26.0 to 27.86	
Age (yrs)			0.847
Mean ± SD	25.0 ± 5.7	24.8 ± 6.0	
Range	(21 to 50)	(21 to 39)	
Sex: *n*, (%)			< 0.001*
Male	290 (16.7%)	218 (37.5%)	
Female	1444 (83.3%)	364 (62.5%)	
Eye: *n*, (%)			0.0004*
Right	830 (47.9%)	328 (56.4%)	
Left	904 (52.1%)	254 (43.6%)	
SE(D)			< 0.001*
Mean ± SD	− 3.3 ± 1.7	− 7.5 ± 2.3	
Range	(− 0.5 to − 10.0)	(− 4.5 to − 10.0)	
*K* min (D):			0.001*
Mean ± SD	43.7 ± 1.5	42.6 ± 1.8	
Range	(40.4 to 49.0)	(39.8 to 45.2)	
*K* max (D)			0.0685
Mean ± SD	44.8 ± 1.6	44.2 ± 1.6	
Range	(41.7 to 49.6)	(41.3 to 46.7)	

*SE* spherical equivalent, *n* number, *yrs* years, *SD* standard deviation, *D* diopter, *K min* minimum keratometric power, *K max* maximum keratometric power

*Statistically significant

**Fig. 4:**
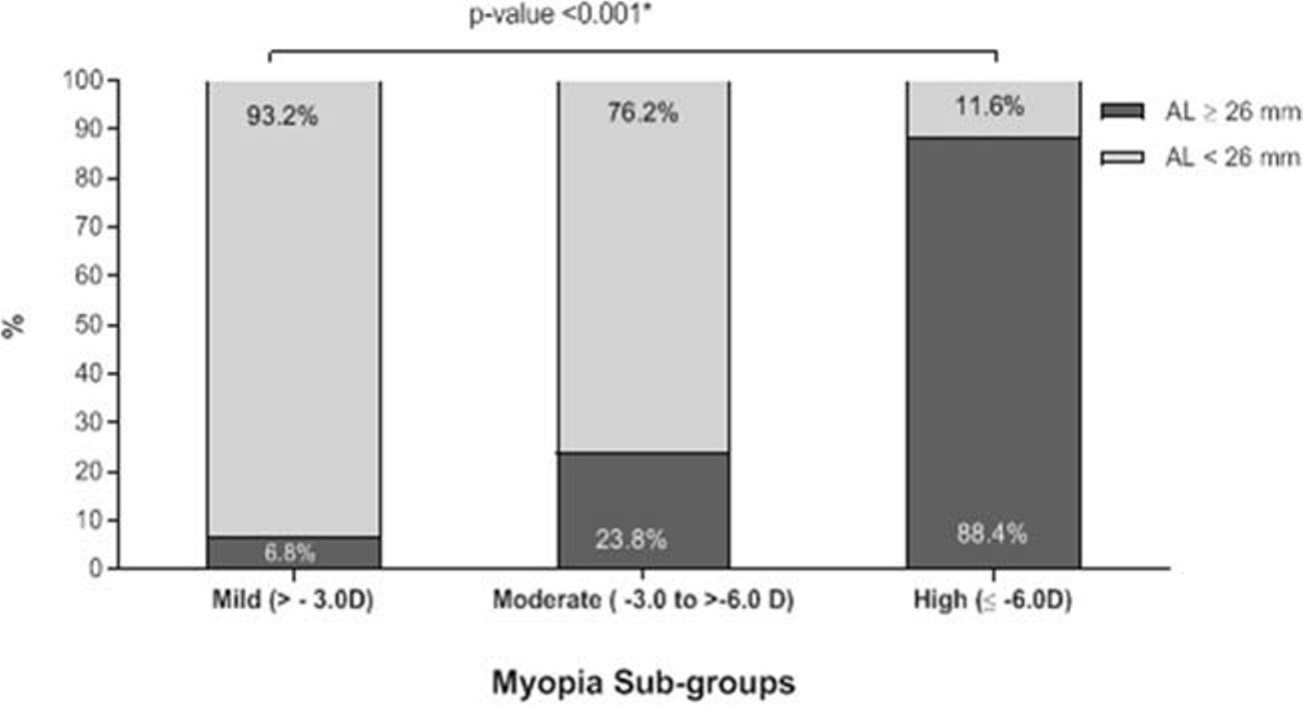
Percentage of patients with myopic regression and axial length ≥ 26 mm

**Fig. 5:**
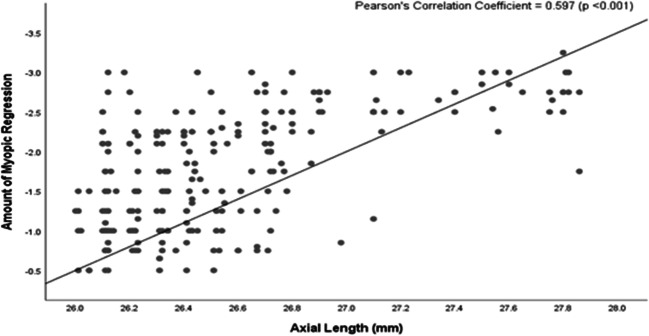
Scatter plot of axial length versus myopic regression

The higher degrees of myopia were predictors of the need for retreatment. In total, 88.4% of the patients with pre-operative high myopia and axial length ≥ 26 mm had myopic regression, 23.8% of the patients with pre-operative moderate myopia and axial length ≥ 26 mm have myopic regression and 6.8% of the patients with pre-operative low myopia, and axial length ≥ 26 mm have myopic regression (Table 5 and Fig. 4). Figure 5 describes the scatter plot of axial length versus myopic regression. It shows that the amount of myopic regression was significantly and positively correlated with the pre-operative axial length (Pearson’s correlation coefficient = 0.597; *P* value < 0.001).

**Table 5:** Percentage of patients with myopic regression and axial length ≥ 26 mm

		Myopia *n* (column %)			Total eyes	*P* value
		Low > − 3.0D	Moderate − 3.0 to > − 6.0D	High ≤ − 6.0D		
Axial length	< 26 mm	1062 (93.2%)	632 (76.2%)	40 (11.6%)	1734	< 0.001*
	≥ 26 mm	78 (6.8%)	198 (23.8%)	306 (88.4%)	582	
	Total eyes	1140	830	346	2316	

*n* number

*Statistically significant

**Fig. 6:**
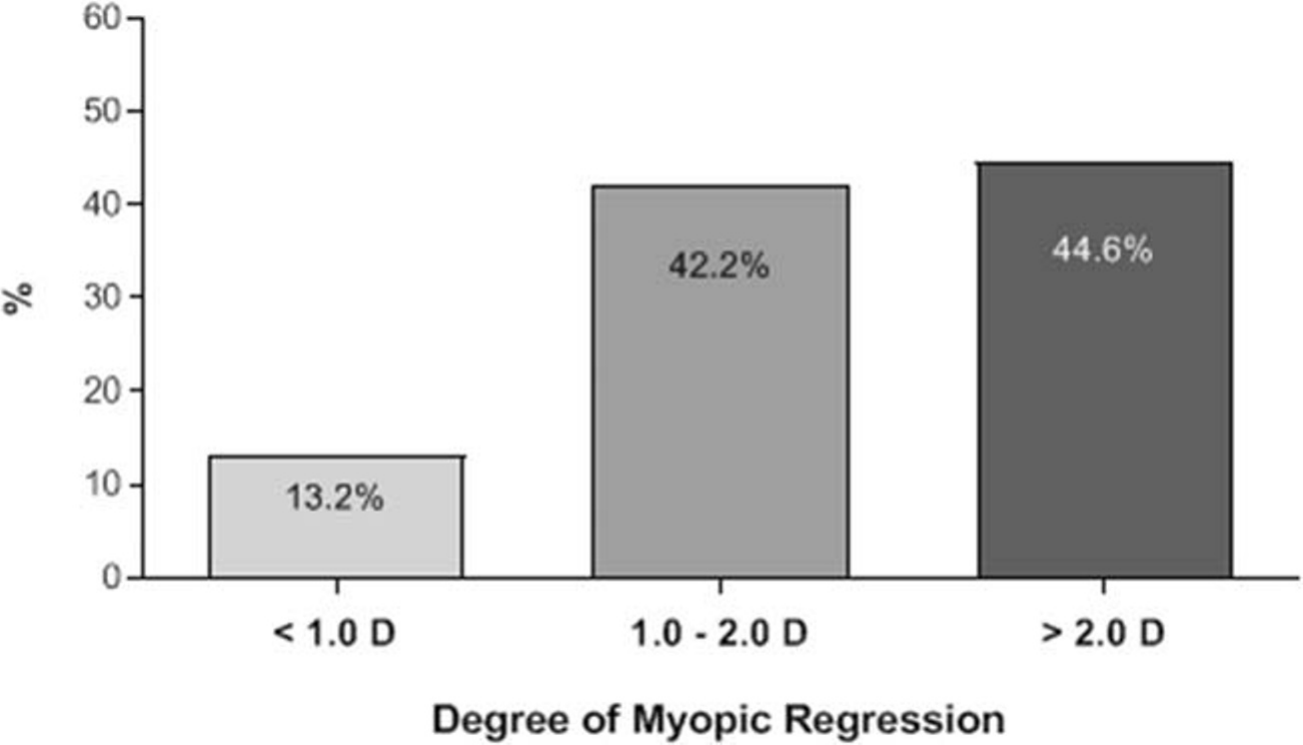
Percentage of post-LASIK myopic regression according to the degree of progression in diopter

In the classification of the eyes with post-LASIK myopic regression regarding the degree of regression, in 77 eyes (13.2%), the myopic regression was < 1.0D, in 246 eyes (42.2%), the regression was 1.0 to 2.0D, and in 260 eyes (44.6%), the regression was > 2.0 D (Fig. 6).

**Fig. 7:**
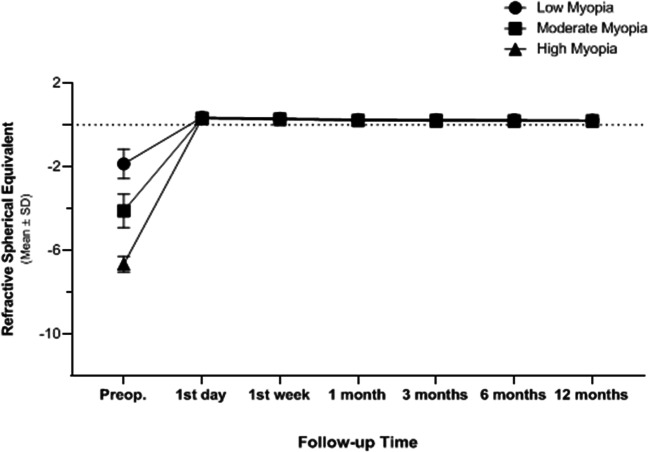
Refractive stability of mean ± SD refractive spherical equivalent between pre-operative and post-operative follow-up time in the 3 myopic sub-groups with stable refraction

The differences between mean *K* values at the 1st month, 6th month, and 12th month post-operative in both eyes with stable refraction and eyes with myopic regression were non-significant (P1 = 0.154 and P2 = 0.970, respectively) (Table 6). The differences between the pre-operative axial length and along the 12-month follow-up time of both eyes with stable refraction and eyes with myopic regression are presented in Table 7. There was a statistically significant increase in the axial length (*p* < 0.001*) along follow-up time in eyes with myopic regression compared to eyes with stable refraction (*P* = 0.580). Mean **±** SD of spherical equivalent (SE) along the 12-month follow-up time of both eyes with stable refraction and after LASIK enhancement in eyes with myopic regression are presented in Table 8. At the end of the follow-up time, eyes with stable refraction had a mean **±** SD of + 0.21 **±** 0.1D, and eyes with myopic regressions had a mean **±** SD of − 0.99 **±** 0.39D. Figure 7 shows the spherical equivalent (SE) pre-operatively and at 1st-day, 1st-week, 1st-month, 3rd-month, 6th-month, and the 12th-month follow-up time after LASIK in the three myopic sub-groups with stable refraction. Spherical equivalent (SE) reduction was statistically significant from the 1st day post-LASIK (*P* **<** 0.001*), with no myopic regression during the remaining follow-up time.

**Table 6:** Mean of *K* reading early post-operative and at the 12-month follow-up time of both eyes with stable refraction and eyes with myopic regression

	Eyes with stable refraction			*P1-value*	Eyes with myopic regression			*P2-value*
	1st month	6th month	12th month		1st month	6th month	12th month	
Mean K (D)	37.6 ± 1.8	37.6 ± 1.75	37.5 ± 1.73	0.153	38.1 ± 1.63	38.12 ± 1.59	38.12 ± 1.58	0.970
Mean ± SD (range)	(34.3 to 42.4)	(34.2 to 42.36)	(34.23 to 42.35)		(35.2 to 41.4)	(35.4 to 41.38)	(35.36 to 41.37)

**Table 7:** Mean of the difference between the pre-operative axial length (mm) and along the 12-month follow-up time of both eyes with stable refraction and eyes with myopic regression

Axial length (mm)	Pre-operative Mean ± SD	1st month Mean ± SD	6th month Mean ± SD	12th month Mean ± SD	*P* value
Eyes with myopic regression	26.6 ± 0.44 (26.0 to 27.86)	26.61 ± 0.45 (26.1 to 27.87)	26.63 ± 0.47 (26.18 to 27.9)	26.9 ± 0.52 (26.3 to 27.92)	< 0.001*
Eyes with stable refraction	24.38 ± 0.73 (22.9 to 25.9)	24.37 ± 0.72 (22.89 to 25.91)	24.37 ± 0.74 (22.91 to 25.91)	24.4 ± 0.73 (22.91 to 25.92)	0.580

*Statistically significant

**Table 8:** Mean of spherical equivalent (SE) along the 12-month follow-up time of both eyes with stable refraction and after LASIK enhancement in eyes with myopic regression

Follow-up	Eyes with stable refraction (D) Mean ± SD				Eyes with myopic regression (enhancement) Mean ± SD
	Low myopia	Moderate myopia	High myopia	Total	
Pre-operative	− 1.86 ± 0.71 (0 to − 3.0)	− 4.11 ± 0.80 (− 3.25 to − 6.0)	− 6.66 ± 0.38 (− 6.26 to − 8.0)	− 2.72 ± 1.42 (0 to − 8.0)	− 2.02 ± 0.62 (− 0.75 to − 3.0)
1st day post-operative	0.36 ± 0.10 (0 to + 0.60)	0.32 ± 0.08 (+ 0.12 to + 0.55)	0.31 ± 0.08 (+ 0.20 to + 0.55)	+ 0.35 ± 0.10 (0 to + 0.60)	+ 0.25 ± 0.09 (+ 0.10 to + 0.50)
1st week post-operative	0.31 ± 0.11 (0.10 to + 0.50)	0.28 ± 0.06 (+ 0.10 to + 0.50)	0.26 ± 0.08 (+ 0.10 to + 0.50)	+ 0.30 ± 0.10 (+ 0.10 to + 0.50)	+ 0.19 ± 0.08 (+ 0.10 to + 0.45)
1st month post-operative	0.26 ± 0.11 (0 to + 0.50)	0.22 ± 0.07 (+ 0.10 to + 0.50)	0.22 ± 0.05 (+ 0.10 to + 0.30)	+ 0.25 ± 0.10 (0 to + 0.50)	+ 0.09 ± 0.08 (0 to + 0.30)
3rd month post-operative	0.25 ± 0.11 (0 to + 0.50)	0.21 ± 0.05 (+ 0.10 to + 0.50)	0.19 ± 0.07 (0 to + 0.30)	+ 0.24 ± 0.10 (0 to + 0.50)	− 0.32 ± 0.25 (0 to − 1.25)
6th month post-operative	0.24 ± 0.11 (0 to + 0.50)	0.20 ± 0.07 (0 to + 0.50)	0.19 ± 0.06 (0 to + 0.30)	+ 0.22 ± 0.10 (0 to + 0.50)	− 0.64 ± 0.29 (− 0.1 to − 1.85)
12th month post-operative	0.22 ± 0.12 (0 to + 0.50)	0.20 ± 0.04 (0 to + 0.50)	0.18 ± 0.07 (0 to + 0.30)	+ 0.21 ± 0.10 (0 to + 0.50)	− 0.99 ± 0.39 (− 0.40 to − 2.5)
*P* value	< 0.001*	< 0.001*	< 0.001*	< 0.001*	< 0.001*

*Statistically significant; *D* diopter

## Discussion

High pre-operative sphere, high astigmatism, corneal steepness, and older age have been well recognized to be associated with retreatment significantly [10–12]. Post-LASIK compensatory epithelial hyperplasia (CEH) is still a debate between researchers, and some report post-LASIK increase in CEH and causes of myopic regression [13–15]; however, others [12, 16] did not report any correlation between CEH and regression.

As far as it is known, this retrospective study is the first to analyse the relationship between the refractive regression and the axial length in myopic patients. It reports that pre-operative high axial length **≥** 26 mm increases the risk of having myopic regression after LASIK. In this study, 25.13% of the patients have an axial length **≥** 26 mm, and all these patients have a myopic regression with varying degrees according to the pre-operative primary treatment. The term myopic regression suggests the loss of effect from the LASIK ablation delivered to match a certain level of myopic refraction at the initial treatment. It suggests a reduction as opposed to the term “progression” which better defines what has happened to these patients. The term LASIK regression would suggest reduction in effect from the laser ablative procedure and not progressive myopia or increase in axial length as deduced by this paper.

Some researchers [17] stated that by the age of 13 years, the axial length of the eye reaches the adult length. So, the eye could not elongate later on. On the other hand, Gudmundsdottir and associates [18] supported that the axial length of the eye changes in adults. They recorded that the mean axial length 23.6 **±** 1.1 mm decreased to 23.2 **±** 1.4 mm along with the 9 years of the study in 50-year-old participants. Fotedar and associates [19] recorded that with age, axial length decreased from a mean of 23.6 to 23.2 mm in the patients aged 85 years along with the 10-year study.

On the other hand, McBrien and Adams [20] investigated the biometric and refractive changes with myopia in adults (average of − 3.74D). They recorded that 48% of the study sample had an increase in the myopia of 0.37D or more during the 2-year time of the study, and this result was because of the elongation of the vitreous body. Fledelius and Goldschmidt [21] recorded a statistically significant increase in the mean of the axial length from 26.7 **±** 1.3 mm at 26 years old to 27.5 **±** 2.1 mm at 54 years old. Some researchers [22, 23] also proposed that, when myopia increases with age, we have to think about the potential increase of axial length in adding to suspect the development of nuclear cataract. These show that in adults, increased myopia with age is because of an increased depth of the vitreous cavity which causes an elongation of the axial length of the eye regardless of the degree of myopia.

Present results strongly suggest that patients with high axial length (**≥** 26 mm) might be increasingly vulnerable to mechanical factors, for example, changing in IOP that can cause stretching of the sclera and increasing the length of the vitreous cavity because of a thin sclera which is considered causing the refractive regression. Also, the relationship between axial length and corneal biomechanics changes after the LASIK was not fully understood and needs further investigations. Wong YZ and Lam AK [24] concluded that patients with high axial length exhibited lower corneal hysteresis than emmetropes. Bueno-Gimeno et al. [25] also recorded that lower levels of corneal hysteresis were associated with longer axial length, and corneal biomechanical properties appeared to be compromised in myopia from an early age, mainly in high myopia. However, some eyes without a posterior staphyloma at the time of the primary treatment may develop it later on overtime.

The present study has some limitations: it is a retrospective study; only successive patients who completed the follow-up period were included (considering the patients who did not complete the follow-up regimen as satisfied patients with their post-LASIK refractive outcome).

In summary, our results support that pre-operative high axial length significantly increases the risk of having myopic regression after LASIK. It should be one of the pre-operative assessment measures for LASIK patients because it could give us an essential clue of post-operative stability and efficacy, not only in high myopic patients but also in low myopic patients as some of them have long axial length. These patients should be closely monitored and followed up regularly.
